# The factors influencing clinical outcomes after leukapheresis in acute leukaemia

**DOI:** 10.1038/s41598-021-85918-8

**Published:** 2021-03-19

**Authors:** Howon Lee, Silvia Park, Jae-Ho Yoon, Byung-Sik Cho, Hee-Je Kim, Seok Lee, Dong-Wook Kim, Nack-Gyun Chung, Bin Cho, Kyoung Bo Kim, Jaeeun Yoo, Dong Wook Jekarl, Hyojin Chae, Jihyang Lim, Myungshin Kim, Eun-Jee Oh, Yonggoo Kim

**Affiliations:** 1 Department of Laboratory Medicine, Seoul St. Mary’s Hospital, College of Medicine, The Catholic University of Korea, 222 Banpo-daero, Seocho-gu, Seoul, 06591 Republic of Korea; 2 Department of Internal Medicine, Seoul St. Mary’s Hospital, College of Medicine, The Catholic University of Korea, Seoul, Republic of Korea; 3 Department of Pediatrics, Seoul St. Mary’s Hospital, College of Medicine, The Catholic University of Korea, Seoul, Republic of Korea; 4 Department of Laboratory Medicine, Incheon St. Mary’s Hospital, College of Medicine, The Catholic University of Korea, Incheon, Republic of Korea; 5 Department of Laboratory Medicine, Eunpyeong St. Mary’s Hospital, College of Medicine, The Catholic University of Korea, Seoul, Republic of Korea; 6 Catholic Genetic Laboratory Center, Seoul St. Mary’s Hospital, College of Medicine, The Catholic University of Korea, Seoul, Republic of Korea; 7 Research and Development Institute for In Vitro Diagnostic Medical Devices of Catholic University of Korea, College of Medicine, The Catholic University of Korea, Seoul, Republic of Korea

**Keywords:** Acute lymphocytic leukaemia, Acute myeloid leukaemia

## Abstract

Leukapheresis is used for the mechanical removal of leukaemic cells in hyperleukocytosis. However, the effectiveness of leukapheresis remains unclear due to selection and confounding factors in the cohorts. We compared the effectiveness of leukapheresis among the subgroups according to either the 2016 World Health Organization classification or the number of cytogenetic abnormalities with a retrospective, single-centre study from January 2009 to December 2018. Acute myeloid leukaemia (AML, n = 212) and acute lymphoblastic leukaemia (ALL, n = 97) were included. The 30-day survival rates (95% confidence interval, 95% CI) for AML and ALL were 86.3% (81.6–90.9%) and 94.8% (90.3–99.2%), respectively. For AML, ‘primary AML with myelodysplasia-related changes’ and ‘AML with biallelic mutation of *CEBPA*’ showed better 30-day survival outcomes (*P* = 0.026) than the other subgroups. A higher platelet count after leukapheresis was associated with better 30-day survival in AML patients (*P* = 0.029). A decrease in blast percentage count after leukapheresis was associated with better 30-day survival in ALL patients (*P* = 0.034). Our study suggested that prophylactic platelet transfusion to raise the platelet count to 50 × 10^9^/L or greater might improve clinical outcome in AML patients undergoing leukapheresis.

## Introduction

Hyperleukocytosis (HL) is defined as a white blood cell (WBC) count equal to or greater than 100 × 10^9^/L in the peripheral blood, but this is somewhat arbitrary because the criteria for WBC counts differ among haematologic diseases. HL is defined as a WBC count of 50–100 × 10^9^/L in acute myeloid leukaemia (AML) and a WBC count equal to or greater than 300 × 10^9^/L in acute lymphoblastic leukaemia (ALL) and chronic lymphocytic leukaemia (CLL)^1–3^. Evidence suggests that a higher WBC count gives rise to a poorer prognosis than a lower WBC count in both children and adults^4–7^. Therefore, emergency strategies to reduce leukaemic cells in the peripheral blood should be taken into consideration.

Leukapheresis is a strategy for immediate reduction of cell mass based on the principle of mechanical removal. There are two theories to explain the effectiveness of leukapheresis. First, by reducing cell mass, mechanical obstruction of small vessels causing leukostatic symptoms can be relieved^8,9^. Second, as leukapheresis recruits leukaemic cells in the S-phase, concomitant chemotherapy (CTx) targeting this phase can become more effective^10,11^.

Leukapheresis is accepted as a second-line therapy (Category II recommendation) in the American Society for Apheresis (ASFA) guidelines^12^. However, some factors make it difficult to assess the effectiveness of leukapheresis. First, as leukapheresis is an invasive procedure, patients with haematologic malignancy might receive prior transfusions; however, transfusion strategies and guidelines differ between institutions. Second, collection volume, anticoagulant volume, and access lines that might affect the effectiveness of leukapheresis also vary between institutions. Third, there is substantial variation in concomitant CTx regimens using hydroxyurea or low-dose CTx agents^13^. These points could be confounding factors when analysing leukapheresis.

Moreover, AML and ALL are further classified and result in various subgroups that have heterogeneous pathophysiologic mechanisms and a range of prognoses^14^. Given these heterogeneities, comparison of these patients without considering subgroups might cause misleading results. The effectiveness of leukapheresis has been inconsistent in previous studies, but the aforementioned factors may have affected this.

The aim of this study was to evaluate the clinical effects of leukapheresis by measuring the difference in parameters before and after leukapheresis, remission rates, and survival rates. We tried to classify each patient using two methods: disease entity based on the 2016 World Health Organization (WHO) classification and number of cytogenetic abnormalities. In addition, we included data on other complete blood cell count (CBC) parameters around the time of leukapheresis.

## Materials and methods

This study is a retrospective, single-centre study performed from January 2009 to December 2018. The electronic medical records were searched for haematologic patients who had bone marrow studies and underwent leukapheresis. Informed consent for study participation was waived by the Institutional Review Board (IRB).

Our protocol was in accordance with the Declaration of Helsinki and approved by the Institutional Review Board (KC20RISI0340) of Seoul St. Mary's Hospital.

### Patients

Most patients with an initial WBC count equal to or higher than 100 × 10^9^/L were treated with repeated leukapheresis until the WBC count decreased below 100 × 10^9^/L during admission. Those patients received induction CTx as soon as the initial leukapheresis was completed, and a bone marrow study revealed haematologic malignancy. In this study, to avoid bias from cumulative leukapheresis procedures or concomitant CTx regimens, we collected only initial leukapheresis data from each patient.

**Figure 1:**
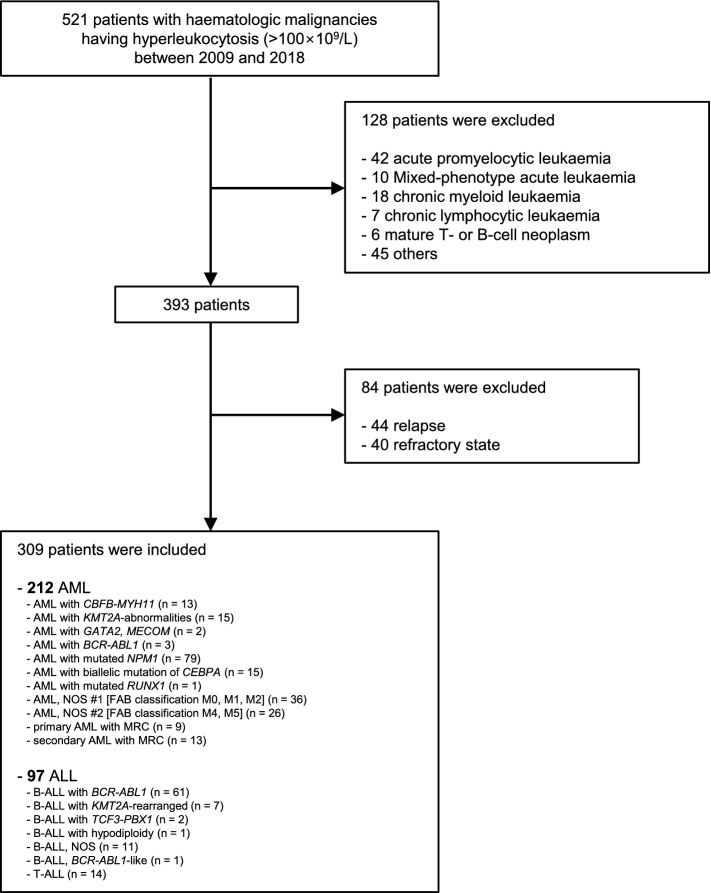
Flow chart. *AML* acute myeloid leukaemia, *ALL* acute lymphoblastic leukaemia, *FAB* French–American–British, *MRC* myelodysplasia-related changes.

Among 521 newly diagnosed acute leukaemia patients who required emergency leukapheresis at our institution from January 2009 to December 2018, we enrolled 309 patients who were diagnosed with AML or ALL. We excluded 128 patients who were diagnosed with another haematologic malignancy as well as 84 patients with previous diagnosis and treatment of leukaemia to avoid bias that can arise from previous medical interventions. Finally, we collected data on 212 AML patients and 97 ALL patients (Fig. 1).

Patients diagnosed before 2016 were reclassified according to the 2016 WHO classification. For reclassification, cytogenetic and molecular findings, including *NPM1* and *CEBPA* genes, were available in our medical records.

Additionally, for AML, the ‘AML not otherwise specified (AML, NOS)’ group was subdivided into two subgroups according to the ordinary French–American–British (FAB) classification, namely, AML M4 and M5, which are reported to be closely related to leukostatic symptoms derived from HL^4,15–19^. ‘AML with myelodysplasia-related changes (AML with MRC)’ is a heterogeneous group that consists of multiple subgroups confirmed by different findings^20^. Therefore, this group was subdivided into two subgroups: ‘primary AML with MRC’, which exhibited de novo multilineage dysplasia or cytogenetic abnormalities, and ‘secondary AML with MRC’, which is derived from a history of myelodysplastic syndrome (MDS) or myelodysplastic/myeloproliferative neoplasm (MDS/MPN).

Irrespective of this subgrouping, all patients were categorized according to the number of cytogenetic abnormalities as having a normal karyotype or having one, two, or three or more cytogenetic abnormalities (a complex karyotype). We used these two approaches in the same cohort to compare the effectiveness of leukapheresis between subgroups.

### Leukapheresis

All leukapheresis procedures were initiated before administration of a cytoreductive regimen. As this study was performed at a single centre, the indications for leukapheresis and the procedure itself were consistent. A central or peripheral venous catheter was placed prior to leukapheresis. The procedure was performed at the bedside using the COBE SPECTRA (Terumo BCT, Lakewood, CO, USA) continuous-flow blood cell separator. Acid citrate dextrose was added for anticoagulation with a citrate-to-blood ratio of 1:13. To avoid a hypocalcaemic effect, 1200 mg of 3% calcium gluconate added to 1 L of Hartmann’s solution was administered throughout the procedure.

All the procedures were continued until approximately 7 L of blood had been processed. Each procedure lasted for a median (range) of 170 min (100–231 min), and the median volume (range) of product collected was approximately 720 mL (300–887 mL), with a coefficient of variation less than 10.0% (Supplementary Figure S1).

Blood cell counts were performed before and after the procedure. Red blood cell (RBC) transfusion should ideally be avoided before leukapheresis^2,3,12,21^. However, to manage possible haemorrhage or bleeding, some cases of anaemia were corrected via packed RBC transfusion before the procedure to reach a haemoglobin (Hb) concentration over 7.0 g/dL^22^. In thrombocytopenia, single-donor platelets (SDPs) were transfused to reach a platelet count over 50 × 10^9^/L before the procedure. Fresh frozen plasma (FFP) was transfused whenever coagulopathy was suspected before the procedure.

### ‘WBC count gap’, ‘Blast absolute count gap’ and ‘Blast percentage gap’

To evaluate the performance of leukapheresis, three parameters before and after the procedure were mainly used. First, ‘WBC count gap (× 10^9^/L)’ indicates the value obtained after subtracting the postleukapheresis WBC count from the preleukapheresis WBC count. Second, the blast absolute count was derived by multiplying the WBC count by the blast percentage. In addition, ‘Blast absolute count gap (× 10^9^/L)’ indicates the value obtaining by subtracting the postleukapheresis blast absolute count from the preleukapheresis blast absolute count. Finally, ‘Blast percentage gap (%)’ indicates the value obtained by subtracting the post-leukapheresis blast percentage from the pre-leukapheresis blast percentage.

### Remission

After the initial leukapheresis procedure, most patients, excluding those who died before intensive care, received induction CTx according to the disease entity confirmed by the bone marrow study. To analyse the association between initial leukapheresis and haematologic complete remission (CR), a follow-up bone marrow study was performed within 2 months for each CTx-treated patient.

### 30-day survival outcome

Most patients were hospitalized for medical treatment until discharge. The effectiveness of leukapheresis was expected to influence early mortality. Data were collected at 30 days from the date of the initial leukapheresis procedure.

### Statistical methods

Categorical variables are presented as frequencies and were compared using the chi square test among the groups. If more than 20% of cells had less than the expected frequency of 5, Fisher's exact test was alternatively used for analysis.

Continuous variables are presented as the median (range) and were compared using Student’s t-test. Comparisons of laboratory data before and after leukapheresis in each group were performed using paired t-tests. Prognostic factors were predicted by univariate and multivariate analysis via the Cox regression method, and all the factors were entered into the model by the backward method. To study the factors that resulted in complete remission, univariate and multivariate analyses using logistic regression were performed for patients who received intensive CTx. In addition, 30-day survival outcomes were analysed using the Kaplan–Meier method and the log-rank test.

The level of statistical significance was set at a p-value of 0.05. Data were analysed using the statistical program SPSS 24 (SPSS Inc., Chicago, IL, USA).

## Results

### Biological and clinical characteristics

Baseline characteristics, concomitant leukostatic symptoms, transfusion records before leukapheresis, and modality setup data for the two groups are described in Table 1.

**Table 1:** Baseline characteristics of acute myeloid leukaemia (AML), acute lymphoblastic leukaemia (ALL) groups.

	AML (n = 212)	ALL (n = 97)
Age	52 (17–87)	42 (11–75)
Sex (male/female)	114/98	45/52
Body weight	64 (26–101)	60 (20–88)
Laboratory data		
WBC (× 10^9^/L), before	164.0 (94.8–429.7)	204.8 (83.8–615.2)
Blasts %(peripheral), before	90 (2–99)	93 (52–99)
RBC (× 10^12^/L), before	2.7 (1.2–4.6)	3.1 (1.5–5.1)
Hb (g/dL), before	8.6 (4.1–15.3)	9.2 (4.4–15.3)
Platelet (× 10^9^/L), before	48.5 (8.0–376)	48.0 (5.0–252)
Cytogenetic abnormalities^a^ (0/1/2/≥ 3)	125/56/15/11	5/37/18/36
Leukostatic symptoms (≥ 1/no)	103/109	62/35
Mental change (yes/no)	1/211	0/97
Headache (yes/no)	34/178	29/68
Impaired vision (yes/no)	2/210	6/91
Fatigue (yes/no)	34/178	18/79
Dizziness (yes/no)	30/182	22/75
Tinnitus (yes/no)	1/211	3/94
Dyspnea (yes/no)	32/180	19/78
Transfusion		
RBC (yes/no)	68/144	20/77
PRC (unit) 1/2/3/4	26/34/3/5	8/10/1/1
Platelet (yes/no)	129/83	62/35
SDP (unit) 1/2/3	99/25/1	41/18/2
PC (unit) 6/12	3/1	1/0
FFP (yes/no)	33/179	5/92
FFP (unit) 1/2/3/4/6	1/8/19/4/1	0/0/3/2/0
Procedures		
Access line		
Peripheral/central/mix	175/10/27	79/4/14
Anti coagulant (ACD-A)	539 (262–839)	539 (95–564)
Removed volume	721 (300–887)	713 (300–825)

*WBC* white blood cell, *RBC* red blood cell, *Hb* haemoglobin, *PRC* packed red blood cells, *SDP* single donor platelets, *PC* platelet concentrates, *FFP* fresh frozen plasma, *ACD-A* acid citrate dextrose-A, *NS* not significant.

^a^Missing data in the AML (5 cases), and the ALL (1 case) groups.

In 212 AML patients, the median WBC count and blast percentage in the peripheral blood were 164.0 (× 10^9^/L) and 90 (%), respectively. The number of patients without cytogenetic abnormalities was greater than that of patients with cytogenetic abnormalities. The number of patients who featured at least one leukostatic symptom was 103, but the presence of any symptoms was not significantly related to CBC parameters (Supplementary Table S2). In addition, the presence of each cluster of designation (CD) marker showed no significant association with the WBC count or blast percentage (Supplementary Table S3).

In 97 ALL patients, the median WBC count and blast percentage in the peripheral blood were 204.8 (× 10^9^/L) and 93 (%), respectively. The patients with cytogenetic abnormalities outnumbered the patients without cytogenetic abnormalities. The patients who featured at least one leukostatic symptom were 62 patients; in particular, the presence of headache was significantly associated with a higher WBC count than the absence of headache, with a median of 218.3 (116.1–584.8) (× 10^9^/L) versus 194.0 (83.8–615.2) (× 10^9^/L) (*P* = 0.029) (Supplementary Table S2). Likewise, no CD markers showed significant associations with the WBC count or blast percentage (Supplementary Table S3).

### ‘WBC count gap’ and ‘blast percentage gap’ in AML and ALL

**Figure 2:**
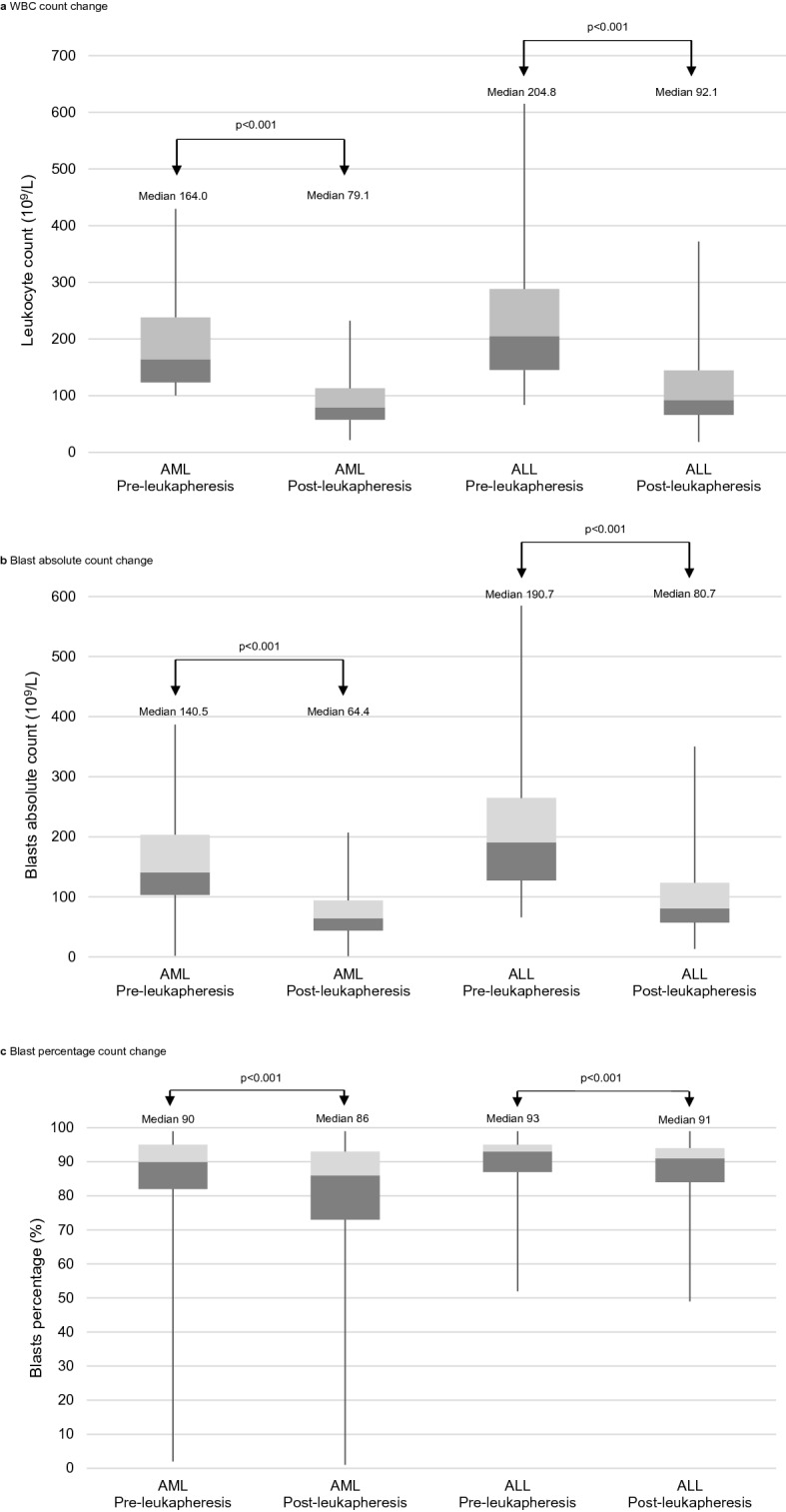
Changes in CBC parameters before and after leukapheresis in the acute myeloid leukaemia (AML) and acute lymphoblastic leukaemia (ALL) groups. Each boxplot represents the range from the 25th to 75th percentile of each group. *CBC* complete blood cell, *WBC* white blood cell.

After initial leukapheresis, the peripheral WBC count significantly decreased in the AML and ALL groups (*P* < 0.001, both) (Fig. 2). The peripheral blast percentages also significantly decreased in the AML and ALL groups (*P* < 0.001, both).

Using the 2016 WHO classification, with the exception of a few subgroups that were too small to be analysed, all disease entities showed a significant reduction in WBC counts after initial leukapheresis (Supplementary Table S4). All subgroups showed a decrease in blast percentage, with or without statistical significance.

With regard to the number of cytogenetic abnormalities, significant reductions in WBC and blast count were observed in all subgroups, with the exception of 6 patients whose initial karyotypes were not identified. In AML, 60.4% (125/207) had a normal karyotype, 27.1% (56/207) had one cytogenetic abnormality, 7.2% (15/207) had two cytogenetic abnormalities, and 5.3% (11/207) had a complex karyotype (Supplementary Table S5). The WBC count was significantly decreased after leukapheresis in all chromosomal states, and the changes in blast percentage by subgroup were as follows: normal, from 90 to 87% (*P* < 0.001); one abnormality, from 90 to 88% (*P* < 0.001); two abnormalities, from 92 to 85% (not significant, NS); and complex, from 87 to 85% (*P* = 0.012). In ALL, 5.2% (5/96) had a normal karyotype, 38.5% (37/96) had one cytogenetic abnormality, 18.8% (18/96) had two cytogenetic abnormalities, and 37.5% (36/96) had a complex karyotype (Supplementary Table S6). The normal karyotype subgroup could not be analysed due to an insufficient number of patients. The WBC count was significantly decreased in all subgroups after leukapheresis. The changes in blast percentage in the subgroups were as follows: normal, from 94 to 87% (not available, NA); one abnormality, from 94 to 90% (*P* = 0.003); two abnormalities, from 94 to 92% (*P* = 0.034); and complex, from 92 to 92% (NS).

### ‘WBC count gap’ and ‘blast percentage gap’ according to each CD marker

In AML, the patients whose blasts were positive for CD14 showed a significantly smaller WBC count gap between procedures than those whose blasts were negative for CD14, as follows: 72.6 (18.3–169.6) (× 10^9^/L) versus 86.0 (11.0–302.2) (× 10^9^/L) (*P* = 0.003) (Supplementary Table S3). This implies that the WBC count of CD14-positive patients seldom decreased compared with that of CD14-negative patients. The patients whose blasts were positive for CD13 or HLA-DR showed significantly more blast gaps, but their differences were small. Other CD markers, including CD2, CD7, CD11c, CD33, CD34, CD56, CD64, CD117 and even cytoplasmic MPO, did not show significant associations with the WBC count gap or blast percentage gap between procedures.

In ALL, no significant association between each CD marker and the above parameters was observed (Supplementary Table S3).

### CR rate

Subgroup analysis for CTx recipients in the AML or ALL group was performed to predict covariates related to CR. Among 161 CTx patients with AML, 107 had CR, and 54 had non-CR.

**Figure 3:**
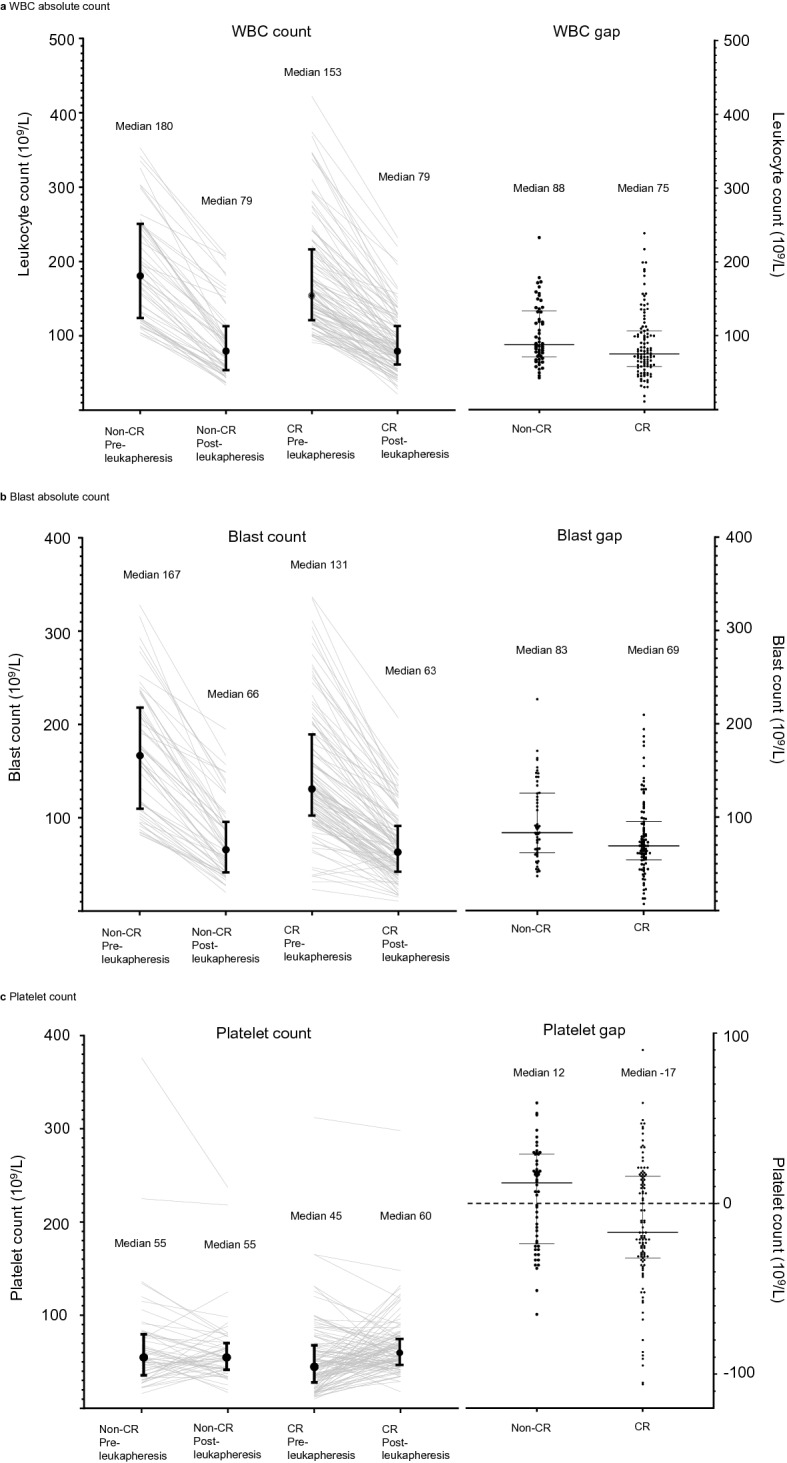
Changes in CBC parameters before and after leukapheresis in the non-CR and CR groups in the acute myeloid leukaemia (AML) group. Each error bar represents the range from the 25th to 75th percentile of each group. *CBC* complete blood cell, *CR* complete remission, *WBC* white blood cell.

The median WBC count gaps for the CR group and non-CR group were 75.2 and 88.2 (× 10^9^/L), respectively (Fig. 3). The median initial blast absolute counts for the CR group and non-CR group were 130.9 and 166.8 (× 10^9^/L), respectively. After leukapheresis, these parameters changed to 63.3 and 65.8 (× 10^9^/L), suggesting that the blast absolute count gap is proportional to the initial blast absolute count. In CR prediction, the smaller the blast absolute count gap was, the higher the probability of CR in our model (Table 2).

**Table 2:** Prediction for complete remission (CR) using univariate and multivariate logistic regression in acute myeloid leukaemia (AML) group.

AML	CR			
	Univariate		Multivariate	
	*P* value	OR (95% CI)	*P* value	OR (95% CI)
WBC (× 10^9^/L), before initial leukapheresis	0.036	0.995 (0.991–1.000)		
WBC gap (× 10^9^/L), between initial leukapheresis	0.023	0.991 (0.984–0.998)		
Blasts absolute (× 10^9^/L), before initial leukapheresis	0.022	0.955 (0.991–0.999)		
Blasts absolute gap (× 10^9^/L), between initial leukapheresis	0.003	0.988 (0.981–0.996)	0.002	0.986 (0.977–0.995)
Platelet gap (× 10^9^/L), between initial leukapheresis	0.002	0.985 (0.976–0.995)	0.004	0.983 (0.971–0.994)
No FLT3 mutation	< 0.001	5.187 (2.563–10.510)	< 0.0001	4.793 (2.335–9.840)

*OR* odds ratio, *CI* confidential interval.

The median initial platelet count was lower in the CR group than in the non-CR group: 45.0 and 55.0 (× 10^9^/L), respectively (*P* = 0.034) (Fig. 3). However, the platelet count became higher in the CR group than in the non-CR group after leukapheresis, mainly due to the effect of platelet transfusion before the procedure. In CR prediction, the smaller the platelet count gap was, the higher the probability of CR (Table 2), which was expected in our model.

In ALL, among 86 CTx patients, 56 had CR, and 30 had non-CR. There were none of the covariates that predicted CR in ALL.

In addition, there were no significant differences based on the number of cytogenetic abnormalities. When we subdivided these chromosome-based data into AML and ALL (Supplementary Tables S7 and S8), there was still no definite association between the CR rate and the number of cytogenetic abnormalities (*P* = 0.470 for the AML group, *P* = 0.393 for the ALL group).

### Early mortality

The 30-day survival rates (95% confidence interval, 95% CI) of the AML and ALL groups were 86.3% (95% CI, 81.6–90.9%) and 94.8% (95% CI, 90.3–99.2%), respectively.

There was a survival difference (*P* = 0.026) among the AML subgroups based on the 2016 WHO classification. All patients in either the ‘AML with *CBFB-MYH11*′ or ‘primary AML with MRC’ subgroups survived. On the other hand, the ‘AML with *KMT2A* abnormalities’ and ‘secondary AML with MRC’ subgroups showed the worst survival outcome, with 30-day survival rates of 80.0% (95% CI, 75.5–84.4%) and 61.5% (95% CI, 57.5–65.4%), respectively (Supplementary Fig. S9). In the ALL group (Supplementary Fig. S10), there was no significant difference among the subgroups (*P* = 0.855), and the 30-day survival rates of ‘B-lymphoblastic leukaemia with *BCR-ABL1*’, ‘B-lymphoblastic leukaemia, not otherwise specified (B-ALL, NOS)’, and ‘T-lymphoblastic leukaemia (T-ALL)’ groups were 95.1% (95% CI, 90.8–99.3%), 90.9% (95% CI, 85.1–96.6%), and 92.9% (95% CI, 87.7–98.0), respectively.

There were no significant differences among the subgroups categorized according to the number of cytogenetic abnormalities. When these subgroups were analysed in the category of either AML or ALL (Supplementary Fig. S11), the result was not significant (*P* = 0.795, 0.389, respectively).

We analysed early mortality and categorized subgroups according to several CBC parameters. The WBC count gap, blast percentage gap, and platelet count around the time of the leukapheresis procedure were studied in the AML and ALL groups. Specific cut-off levels for each parameter were generated by receiver operating characteristic (ROC) curves.

**Figure 4:**
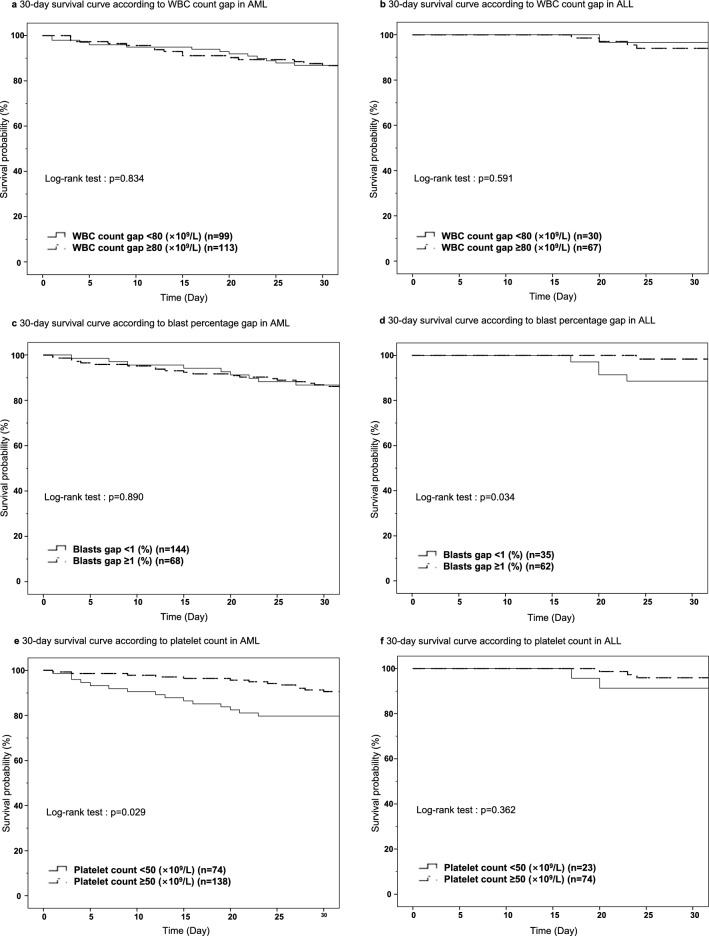
Survival among the subgroups according to CBC parameters in the acute myeloid leukaemia (AML) and acute lymphoblastic leukaemia (ALL) groups. *CBC* complete blood cell, *WBC* white blood cell.

In AML, there was no significant difference when groups were subdivided by WBC count gap with a cut-off value of 80 × 10^9^/L (*P* = 0.834) or blast percentage gap with a cut-off value of 1% (*P* = 0.890) (Fig. 4a, c). However, there were significant findings when the groups were subdivided by platelet count with a cut-off value of 50 × 10^9^/L (*P* = 0.029) (Fig. 4e). A higher platelet count around the time of leukapheresis was correlated with a higher 30-day survival rate of 89.9% (95% CI, 83.5–94.3%), compared to 79.7% (95% CI, 75.2–84.1%) for patients with a lower platelet count.

In ALL, no significant findings were observed when the groups were subdivided by the WBC count gap with a cut-off value of 80 × 10^9^/L (*P* = 0.591) (Fig. 4b). When subdivided the groups were subdivided by blast percentage gap with a cut-off value of 1%, a higher blast percentage gap was correlated with a favourable prognosis, with a 30-day survival rate of 98.4% (95% CI, 95.9–100%), compared to 88.6% (95% CI, 82.2–94.9%) for patients with a lower blast percentage gap (*P* = 0.034) (Fig. 4d). There were no significant differences according to platelet count (*P* = 0.362) (Fig. 4f).

Univariate and multivariate Cox regression analyses were performed (Table 3). In AML, the groups that were not treated with CTx or exhibited an absence of CD117 showed unfavourable 30-day survival, with hazard ratios of 8.47 (3.584–18.076) and 5.68 (2.638–12.230), respectively (*P* < 0.001, both). In ALL, a higher blast absolute gap or lower blast absolute count after leukapheresis predicted a favourable 30-day prognosis, with a hazard ratio of 0.869 (0.760–0.994), and this difference was statistically significant (*P* = 0.040). Leukapheresis frequency predicted unfavourable 30-day survival, with a hazard ratio of 3.994 (1.413–11.920), and this difference was statistically significant (*P* = 0.009).

**Table 3:** Prediction for 30-day survival using univariate and multivariate logistic regression in acute myeloid leukaemia (AML), acute lymphoblastic leukaemia (ALL) groups.

AML	30-day survival in AML				30-day survival in ALL			
	Univariate		Multivariate		Univariate		Multivariate	
	*P* value	HR (95% CI)	*P* value	HR (95% CI)	*P* value	HR (95% CI)	*P* value	HR (95% CI)
No CTx	< 0.001	6.636 (3.164–13.910)	< 0.001	8.470 (3.584–18.076)	NS			
CD14	0.024	2.520 (1.132–5.611)			NS			
No CD117	< 0.001	4.702 (2.208–10.010)	< 0.001	5.680 (2.638–12.230)	NS			
No CR	< 0.001	28.150 (3.701–214.100)	0.002	25.820 (3.327–200.400)	NS			
Cytoplasmic-MPO	0.006	0.329 (0.148–0.732)			NS			
Leukapheresis frequency (n)	0.033	1.380 (1.026–1.857)			0.008	3.297 (1.364–7.972)	0.009	3.994 (1.413–11.920)
Karyotype abnormalities (n)	0.011	1.477 (1.092–1.997)			NS			
Blasts absolute gap (peripheral), between initial leukapheresis	NS				0.043	0.882 (0.780–0.996)	0.04	0.869 (0.760–0.994)
Platelet (× 10^9^/L), after initial leukapheresis	0.015	0.976 (0.976–0.995)			NS			
FLT3 mutation	< 0.001	2.541 (1.580–4.087)			NS			

*HR* hazard ratio, *CTx* chemotherapy, *NS* not significant.

## Discussion

There have been some studies on the effectiveness of leukapheresis in leukaemia patients. Giles et al.^23^ and Bug et al.^24^ reported that leukapheresis reduced early mortality. In contrast, Chang et al. reported that leukapheresis had no significant influence on early mortality^25^, and Malkan et al. found a much higher death rate in the leukapheresis group^26^. Moreover, Kuo et al. demonstrated equivalent early survival rates between leukapheresis and hydroxyurea^27^.

Oberoi et al. pointed out that some previous studies were at high risk of selection and confounding bias^28^. A randomized controlled trial has not been conducted, but analytical studies with matched control groups have been used in an attempt to minimize bias. Nan et al. found that leukapheresis was associated with a significantly lower early mortality rate than a matched control group^29^. However, Stahl et al. were unable to detect an early mortality benefit from leukapheresis compared with matched controls^30^.

All studies mentioned above focused on AML. Other types of leukaemia may also feature HL, so leukapheresis should be taken into consideration. Choi et al. performed a propensity score-matched study for AML and ALL and did not observe significant differences in early mortality^31^.

Even in studies using a matched control group, the results have remained heterogeneous, making it difficult to perform routine leukapheresis. We posit that this heterogeneity is caused by multiple factors, including differences in detailed disease entities specified by the 2016 WHO classification as well as the number of cytogenetic abnormalities.

In our study, some patients did not receive chemotherapy, as they died before it could be administered. Excluding them brought about a shortage of patients in each subgroup, so we did not analyse remission rates by the 2016 WHO classification. There was no significant correlation between the remission rate and the number of cytogenetic abnormalities. Some studies have mentioned the association between the number of cytogenetic abnormalities and CR rates, which are inversely proportional to each other in AML^32–35^ and not significant in ALL^36^. However, in our study, the subgroup with 3 or more cytogenetic abnormalities in AML and ALL showed CR rates indistinguishable from those of subgroups with fewer cytogenetic abnormalities after leukapheresis (Supplementary Tables S7 and S8).

We regarded the 30-day survival rate as the most reliable outcome. Considering the 2016 WHO classification, we noticed two findings in AML (Supplementary Fig. S9). First, there were no deaths in the ‘AML with *CBFB-MYH11*′ or ‘primary AML with MRC’ subgroup. According to the 2017 European LeukemiaNet (ELN) risk stratification, all ‘AML with *CBFB-MYH11*’ patients in our cohort are considered favourable, whereas ‘primary AML with MRC’ patients have either intermediate or adverse risk^37^. Second, ‘AML with biallelic mutation of *CEBPA*’ had the highest WBC count of 241.9 × 10^9^/L (130.9–346.5) among all AML subgroups (Supplementary Table S4). However, the 30-day survival rate was 93.3%, higher than all other subgroups except ‘AML with *CBFB-MYH11*’ and ‘primary AML with MRC’. These data might imply that ‘primary AML with MRC’ and ‘AML with biallelic mutation of *CEBPA*’ may benefit more from leukapheresis than other subgroups.

In addition to disease entity, we attempted to correlate clinical outcomes with CBC parameters in AML and ALL. Intuitively, a wider range of WBCs and blast absolute count gaps after leukapheresis seemed to be related to higher CR rates. However, AML in our study, with a wider range of blast absolute count gaps, showed an odds ratio less than one for CR (Table 2). This could be interpreted as a negative effect of leukapheresis for establishing CR, but the authors thought that the reason originated from the difference in the initial blast absolute count rather than the blast absolute count gap. As shown in Fig. 3b, the non-CR group had a higher initial blast absolute count, so a wider blast absolute count gap was observed after leukapheresis, with a correlation coefficient of 0.739 (*P* < 0.001). Giles et al. reported that HL significantly decreased the CR rate^23^, and Chen et al. reported that HL required more chemotherapy cycles than non-HL to achieve CR^38^. Therefore, performing leukapheresis and reducing WBCs or blasts might still be more important than a greater number of WBCs or blast reduction efficiency for CR in AML. In ALL, a higher blast absolute count gap did not predict favourable CR rates. There might have been several reasons, but the authors suggest that this result is derived from the initial blast absolute count. As shown in Fig. 2b, a much higher initial blast absolute count and a relatively wider range of blast absolute count gaps are observed in ALL than in AML. Even if blast reduction by leukapheresis would also be helpful for achieving CR in ALL, wider dispersion of blast cell counts in ALL might result in an insignificant *P*-value in regression analysis.

A larger reduction in WBC count by initial leukapheresis did not ensure a better survival rate (Fig. 4a, b). However, the 30-day survival rate according to blast percentage gap was significant only in ALL (Fig. 4d). The percentage is the relative parameter, and a greater than 1% blast percentage gap indicates a decrease in blast percentage among blood cells after leukapheresis. This could be interpreted in various ways. The authors conceived that reducing haematocrit in the collection port may lead to efficient blast removal while minimizing the waste of other blood cells, as blasts are positioned in the upper layer in the buffy coat.

Meta-analysis by Oberoi et al.^28^, as well as recently published studies by Stahl et al.^30^ and Bewersdorf et al.^39^, showed that there were no 30-day survival benefits after leukapheresis. The difference between previous studies and this study is that transfusion before leukapheresis was considered and platelet supplementation was a covariate that influenced clinical outcomes. RBC or Hb around the time of leukapheresis did not show significant outcome differences, but platelet count was favourably associated with CR and 30-day survival in AML (Table 2 and Fig. 4e). Previously, platelet count was reported to be decreased after leukapheresis^40–42^, and prophylactic platelet transfusion is recommended before central venous catheter insertion for patients with platelet counts less than 20 × 10^9^/L^43,44^. However, there are no guidelines for prophylactic platelet transfusion for leukapheresis, and there is wide variation across regional and institutional guidelines. Platelets are related with immune function by activating dendritic cells, secreting granules that activate T-cells, influencing T-/B-cell response, supporting innate and adaptive immunity, influencing leukocytes, and containing Toll-like receptor transcripts^45–47^. As platelets are related to coagulation and immunologic functions, replenishment of platelets by transfusion in the CR group might have been related with favourable prognosis. In our study, a platelet count higher than 50 × 10^9^/L prior to leukapheresis in AML was associated with CR (Table 2) and favourable 30-day survival. Additional data and evidence are required for administration of platelet as a therapeutic modality.

Our study has several limitations. Comparison groups and subgroups without leukapheresis were not included, as most patients with an initial WBC count equal to or higher than 100 × 10^9^/L routinely underwent leukapheresis at our institution. The post-CBC sampling time interval from leukapheresis ranged from less than 1 to 24 h, which might cause variation in the results. Nevertheless, our study offers new insight into differences between disease entities based on modern concepts. Moreover, haematologic conditions prior to leukapheresis can be the sole rationale for clinical decision making.

In summary, the authors compared the effectiveness of leukapheresis among subgroups according to either the 2016 WHO classification or the number of cytogenetic abnormalities. All subgroups showed a significant reduction in WBC count after leukapheresis. In the 2016 WHO classification, the ‘primary AML with MRC’ and ‘AML with biallelic mutation of *CEBPA*’ subgroups showed better early survival outcomes than other AML subgroups. The statistical model showed that the blast absolute count gap was related to CR in AML. This suggested that leukapheresis may have an indirect positive impact on leukaemia treatment. Moreover, platelet count around the procedure was favourably associated with CR and the 30-day survival rate in AML. Therefore, the tumour burden that may inhibit CR could be removed via leukapheresis with transfusion of enough platelets.

## Supplementary Information

Supplementary Information 1.

Supplementary Information 2.

Supplementary Information 3.

Supplementary Information 4.

Supplementary Information 5.

Supplementary Information 6.

Supplementary Information 7.

Supplementary Information 8.

Supplementary Information 9.

Supplementary Information 10.

Supplementary Information 11.
