# Supplementary material for: The factors influencing clinical outcomes after leukapheresis in acute leukaemia
Source: Sci Rep. 2021 Mar 19;11:6426. doi: 10.1038/s41598-021-85918-8 (PMC7979875; doi:10.1038/s41598-021-85918-8)
Supplement: Supplementary file 1 — Supplementary Information 1. [file 41598_2021_85918_MOESM1_ESM.pptx]

## Slide 1
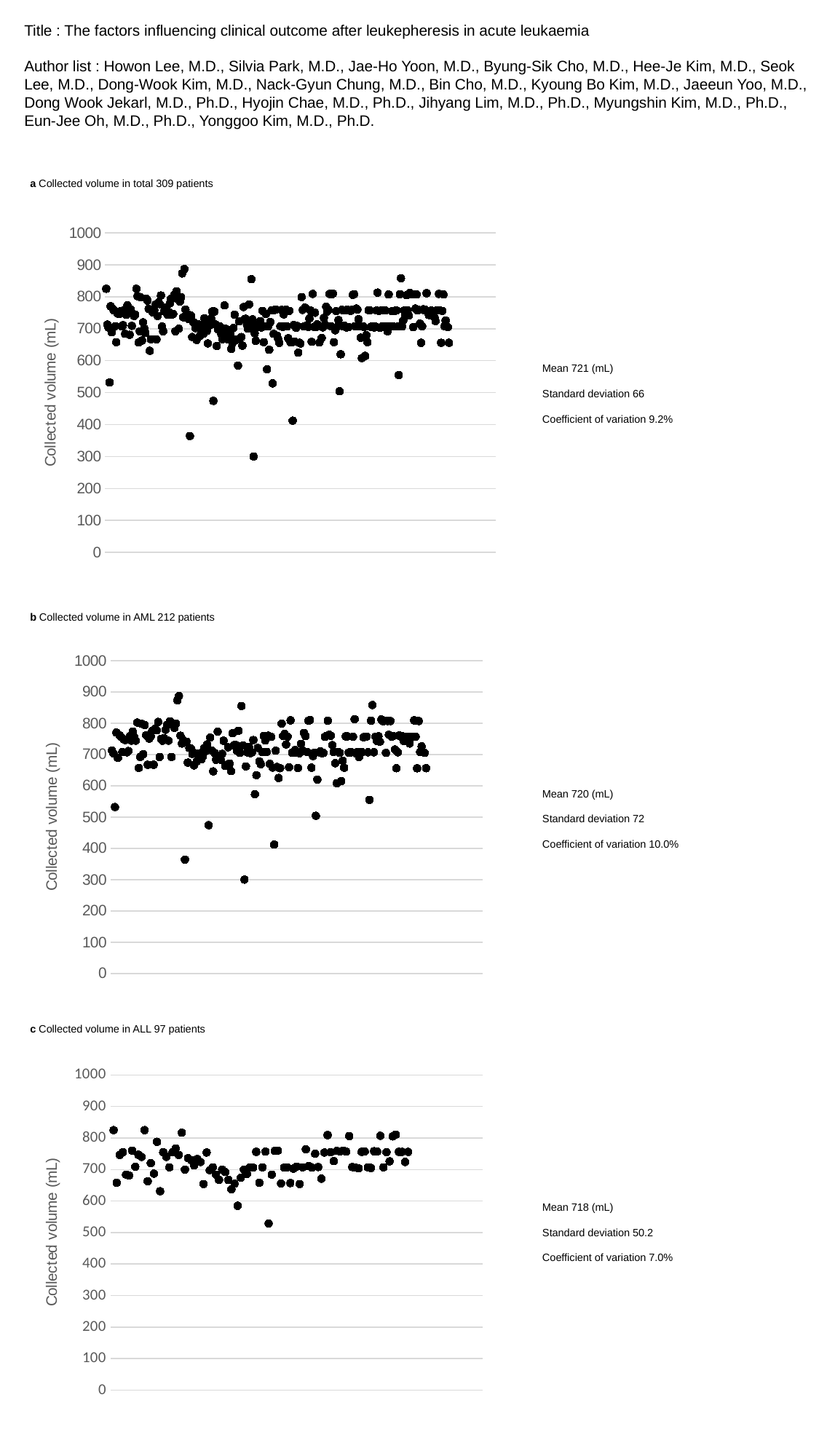

Title : The factors influencing clinical outcome after leukepheresis in acute leukaemia
Author list : Howon Lee, M.D., Silvia Park, M.D., Jae-Ho Yoon, M.D., Byung-Sik Cho, M.D., Hee-Je Kim, M.D., Seok Lee, M.D., Dong-Wook Kim, M.D., Nack-Gyun Chung, M.D., Bin Cho, M.D., Kyoung Bo Kim, M.D., Jaeeun Yoo, M.D., Dong Wook Jekarl, M.D., Ph.D., Hyojin Chae, M.D., Ph.D., Jihyang Lim, M.D., Ph.D., Myungshin Kim, M.D., Ph.D., Eun-Jee Oh, M.D., Ph.D., Yonggoo Kim, M.D., Ph.D.
a Collected volume in total 309 patients
### Chart
| Category | |
|---|---|Mean 721 (mL)
Standard deviation 66
Coefficient of variation 9.2%
b Collected volume in AML 212 patients
### Chart
| Category | 제거량 |
|---|---|Mean 720 (mL)
Standard deviation 72
Coefficient of variation 10.0%
c Collected volume in ALL 97 patients
### Chart
| Category | |
|---|---|Mean 718 (mL)
Standard deviation 50.2
Coefficient of variation 7.0%
