# Supplementary material for: The factors influencing clinical outcomes after leukapheresis in acute leukaemia
Source: Sci Rep. 2021 Mar 19;11:6426. doi: 10.1038/s41598-021-85918-8 (PMC7979875; doi:10.1038/s41598-021-85918-8)
Supplement: Supplementary file 9 — Supplementary Information 9. [file 41598_2021_85918_MOESM9_ESM.pptx]

## Slide 1
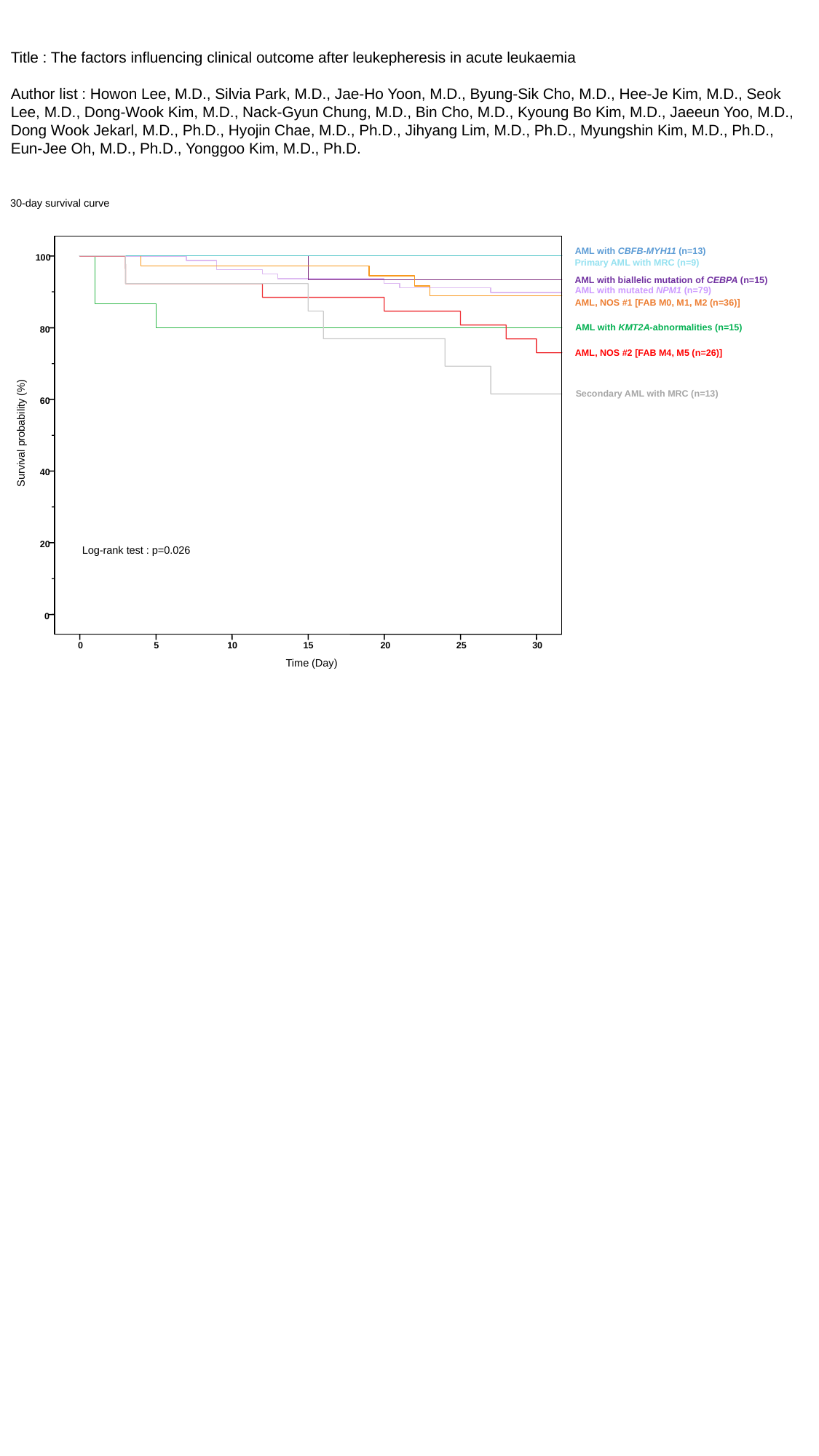

Title : The factors influencing clinical outcome after leukepheresis in acute leukaemia
Author list : Howon Lee, M.D., Silvia Park, M.D., Jae-Ho Yoon, M.D., Byung-Sik Cho, M.D., Hee-Je Kim, M.D., Seok Lee, M.D., Dong-Wook Kim, M.D., Nack-Gyun Chung, M.D., Bin Cho, M.D., Kyoung Bo Kim, M.D., Jaeeun Yoo, M.D., Dong Wook Jekarl, M.D., Ph.D., Hyojin Chae, M.D., Ph.D., Jihyang Lim, M.D., Ph.D., Myungshin Kim, M.D., Ph.D., Eun-Jee Oh, M.D., Ph.D., Yonggoo Kim, M.D., Ph.D.
30-day survival curve
AML with CBFB-MYH11 (n=13)
Primary AML with MRC (n=9)
100
AML with biallelic mutation of CEBPA (n=15)
AML with mutated NPM1 (n=79)
AML, NOS #1 [FAB M0, M1, M2 (n=36)]
AML with KMT2A-abnormalities (n=15)
80
AML, NOS #2 [FAB M4, M5 (n=26)]
Survival probability (%)
Secondary AML with MRC (n=13)
60
40
Log-rank test : p=0.026
20
0
0
5
10
15
20
25
30
Time (Day)
