# Supplementary material for: The factors influencing clinical outcomes after leukapheresis in acute leukaemia
Source: Sci Rep. 2021 Mar 19;11:6426. doi: 10.1038/s41598-021-85918-8 (PMC7979875; doi:10.1038/s41598-021-85918-8)
Supplement: Supplementary file 10 — Supplementary Information 10. [file 41598_2021_85918_MOESM10_ESM.pptx]

## Slide 1
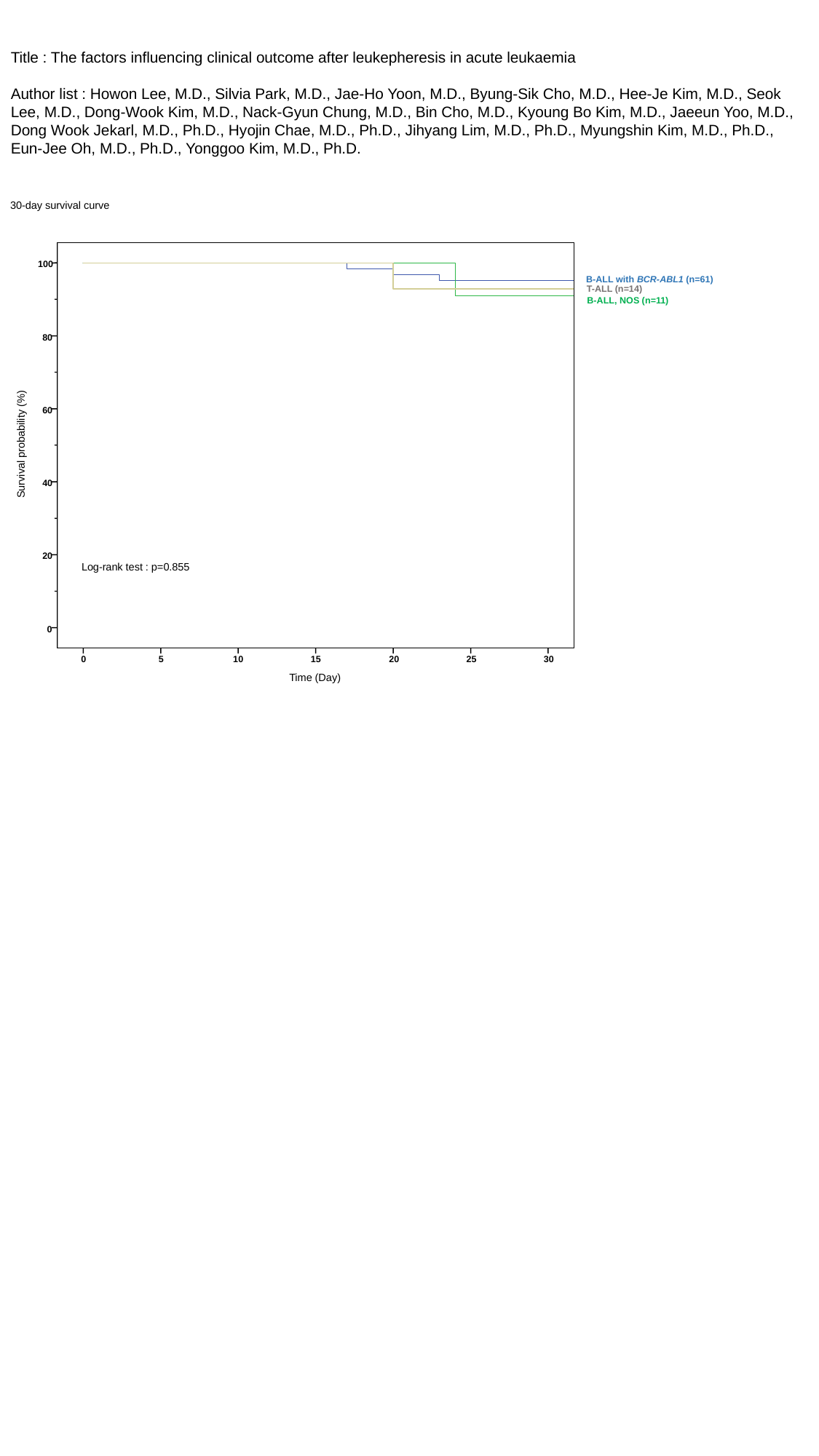

Title : The factors influencing clinical outcome after leukepheresis in acute leukaemia
Author list : Howon Lee, M.D., Silvia Park, M.D., Jae-Ho Yoon, M.D., Byung-Sik Cho, M.D., Hee-Je Kim, M.D., Seok Lee, M.D., Dong-Wook Kim, M.D., Nack-Gyun Chung, M.D., Bin Cho, M.D., Kyoung Bo Kim, M.D., Jaeeun Yoo, M.D., Dong Wook Jekarl, M.D., Ph.D., Hyojin Chae, M.D., Ph.D., Jihyang Lim, M.D., Ph.D., Myungshin Kim, M.D., Ph.D., Eun-Jee Oh, M.D., Ph.D., Yonggoo Kim, M.D., Ph.D.
30-day survival curve
100
B-ALL with BCR-ABL1 (n=61)
T-ALL (n=14)
B-ALL, NOS (n=11)
80
Survival probability (%)
60
40
20
Log-rank test : p=0.855
0
0
5
10
15
20
25
30
Time (Day)
