# Supplementary material for: The factors influencing clinical outcomes after leukapheresis in acute leukaemia
Source: Sci Rep. 2021 Mar 19;11:6426. doi: 10.1038/s41598-021-85918-8 (PMC7979875; doi:10.1038/s41598-021-85918-8)
Supplement: Supplementary file 11 — Supplementary Information 11. [file 41598_2021_85918_MOESM11_ESM.pptx]

## Slide 1
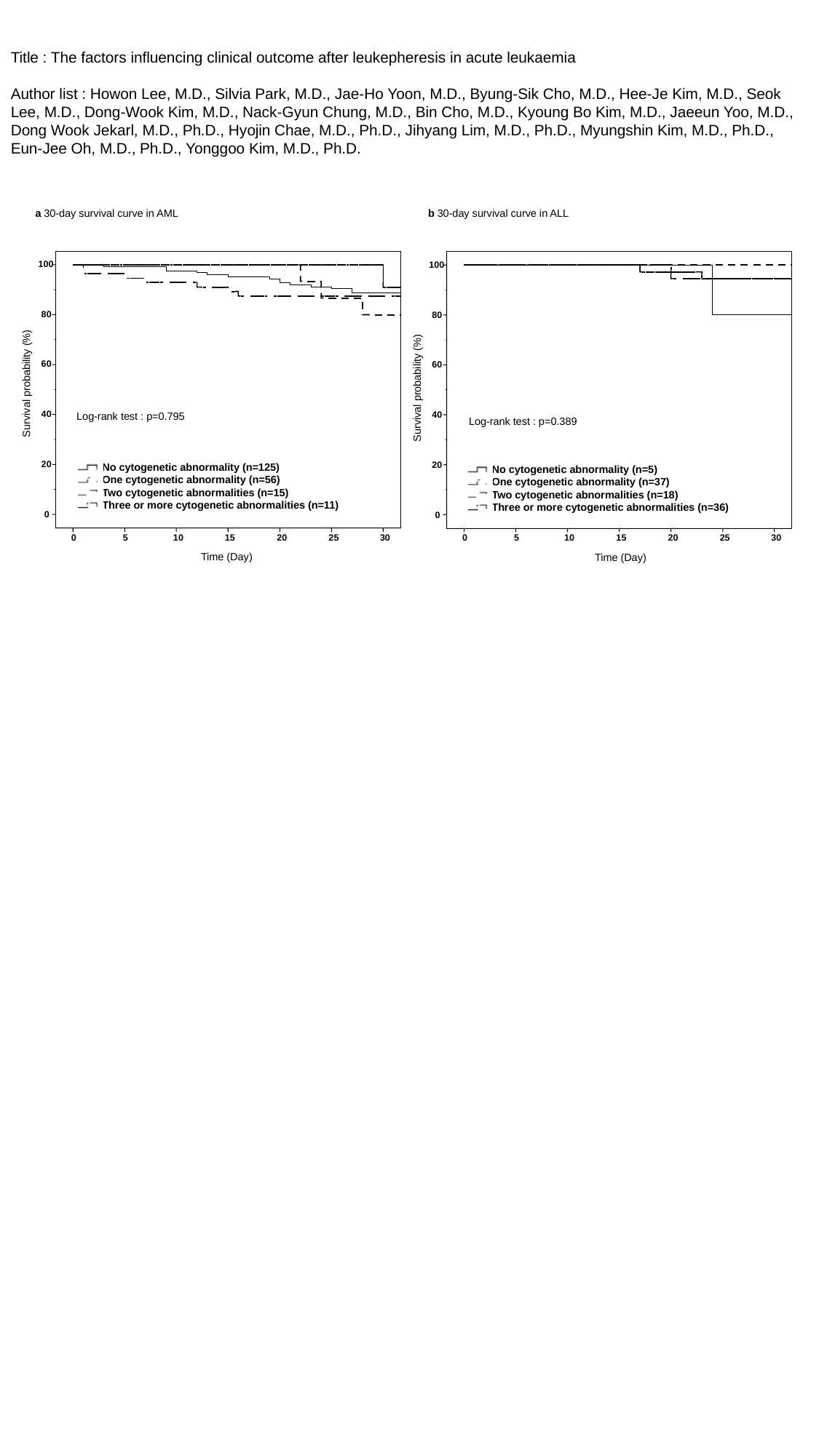

Title : The factors influencing clinical outcome after leukepheresis in acute leukaemia
Author list : Howon Lee, M.D., Silvia Park, M.D., Jae-Ho Yoon, M.D., Byung-Sik Cho, M.D., Hee-Je Kim, M.D., Seok Lee, M.D., Dong-Wook Kim, M.D., Nack-Gyun Chung, M.D., Bin Cho, M.D., Kyoung Bo Kim, M.D., Jaeeun Yoo, M.D., Dong Wook Jekarl, M.D., Ph.D., Hyojin Chae, M.D., Ph.D., Jihyang Lim, M.D., Ph.D., Myungshin Kim, M.D., Ph.D., Eun-Jee Oh, M.D., Ph.D., Yonggoo Kim, M.D., Ph.D.
b 30-day survival curve in ALL
a 30-day survival curve in AML
100
Survival probability (%)
80
60
Log-rank test : p=0.795
40
No cytogenetic abnormality (n=125)
One cytogenetic abnormality (n=56)
Two cytogenetic abnormalities (n=15)
Three or more cytogenetic abnormalities (n=11)
20
0
0
5
10
15
20
25
30
Time (Day)
100
80
Survival probability (%)
60
40
Log-rank test : p=0.389
No cytogenetic abnormality (n=5)
One cytogenetic abnormality (n=37)
Two cytogenetic abnormalities (n=18)
Three or more cytogenetic abnormalities (n=36)
20
0
0
5
10
15
20
25
30
Time (Day)
